# Enteric Methane Emission in Livestock Sector: Bibliometric Research from 1986 to 2024 with Text Mining and Topic Analysis Approach by Machine Learning Algorithms

**DOI:** 10.3390/ani14213158

**Published:** 2024-11-04

**Authors:** Chiara Evangelista, Marco Milanesi, Daniele Pietrucci, Giovanni Chillemi, Umberto Bernabucci

**Affiliations:** 1 Department for Innovation in Biological, Agro-Food and Forest Systems (DIBAF), University of Tuscia, 01100 Viterbo, Italy; marco.milanesi@unitus.it (M.M.); daniele.pietrucci@unitus.it (D.P.); gchillemi@unitus.it (G.C.); 2 Department of Agriculture and Forest Sciences (DAFNE), University of Tuscia, 01100 Viterbo, Italy; bernab@unitus.it

**Keywords:** ruminants, enteric methane, text mining, topic analysis, machine learning

## Abstract

Methane (CH_4_) from livestock, particularly enteric CH_4_ emission (EME), is one contributor to greenhouse gas emissions and climate change. This review analyzed 1294 scientific abstracts on EME in ruminants from 1986 to May 2024, using Scopus^®^ data. Descriptive statistics, text mining, and topic analysis were performed. Publications on EME have risen significantly since 2005, with the *Journal of Dairy Science* being the most frequent publisher. Most studies (82.1%) were original research, with Northern Hemisphere countries leading in publication numbers. The most frequent terms were “milk”, “cow”, and “diet”, while key research topics included greenhouse gas emissions from livestock, diet composition, and prediction models. Despite progress, some areas like CH_4_ emission from animals need further investigation.

## Simple Summary

Methane from livestock is a contributor to greenhouse gases, which are responsible for climate change. A large part of this methane comes from the digestive processes of ruminants. This study looked at how research on methane emissions from livestock has changed over time. By examining 1294 studies published between 1986 and 2024, this review identified key trends and topics in the field. This study found that research in this area has increased significantly since 2005, with most studies coming from Europe and North America, where livestock farming is widespread. The research topics focused on ways to reduce emissions, such as changing animal diets and improving farm practices. One of the most important findings is that while progress has been made, more research is needed to better understand and reduce methane emissions. This knowledge could help in reducing the environmental impact of livestock farming, leading to more sustainable food production and helping to fight climate change.

## 1. Introduction

**Figure 1:**
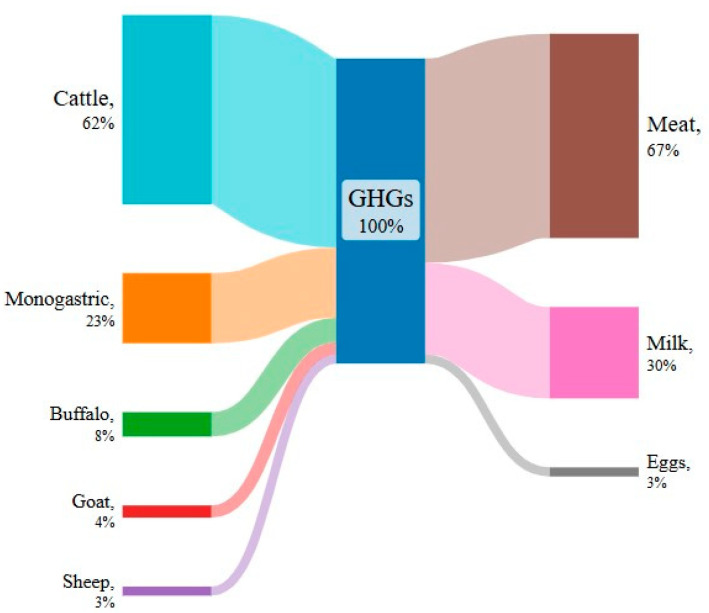
Sankey diagram of GHG emission sources in 2015 by species and products, expressed as a percentage (based on GLEAM 3. FAO [3]). Monogastric species comprise pigs 14% and chickens 9%.

Methane (CH_4_), together with carbon dioxide (CO_2_) and nitrous oxide (N_2_O), are the main greenhouse gases (GHGs) produced by the livestock sector. Greenhouse gas concentrations contribute to climate change and are responsible for the increase in temperature and water scarcity [1]. Compared to pre-industrial levels, the global temperature has increased by 1.5 °C [2], and changes in the natural hydrological cycle of water are occurring, such as increases in intense precipitation, variations in the quantity and seasonal distribution of precipitation, increases in sea levels in coastal communities, increased evapotranspiration, and decreased soil moisture [1]. According to the FAO [3], livestock agrifood systems are responsible for 12% of all anthropogenic GHG emissions, corresponding to 6.2 Gt CO_2_ equivalent emissions (data referring to 2015). Total CH_4_ produced by the livestock sector represents 54%, 31% is CO_2_, and 15% is N_2_O (data referring to 2015, FAO [3]). Methane seems to have the greatest impact on the GHGs produced by the livestock sector since it represents a major share of the GHGs, and the effect on global warming of CH_4_ is approximately 28 times that of CO_2_ [4]. Emissions of GHGs from the livestock sector can occur both directly (through enteric fermentation and manure) and indirectly (through processing, fertilization, feed production, etc.) [4]. Methane produced directly by manure represents 7.8% of the total CH_4_ derived from the livestock sector, while the CH_4_ derived from ruminal fermentations, called enteric CH_4_, is equal to 46% of the total CH_4_ emissions [3]. Significant variations in emission levels and/or intensity are noted among different species, geographical areas, and production systems [5]. According to an FAO report [3], of the six species of livestock animals responsible for producing GHGs, 62% comes from cattle, 8% from buffaloes, 4% from goats, 3% from sheep, and 23% from monogastric animals (pigs and poultry) (Figure 1). Considering the production system, two-thirds of GHG emissions is associated with meat production (67%), followed by milk production (30%) and egg production (3%). A distinction must be made in ruminants: the greater production of CH_4_ is derived from ruminal fermentation, whereas in monogastric systems, the main contributors are feed production, land use change, and manure management. 

The demand for animal products (meat, milk, and eggs) is projected to grow by 20% by 2050 compared to 2020 levels due to the world’s population growth and increasing prosperity [3]. This increased demand leads to an inevitable increase in GHG emissions from the livestock sector to satisfy the growing demand for proteins. Implementing sustainable methods is essential for achieving reduced emissions and lessening the negative effects of livestock systems on the environment. Methane emissions have a shorter average lifespan in the atmosphere than CO_2_ (~12 years versus hundreds of years, respectively), which makes it an attractive improvement target for short-term gains in global warming abatement [6]. Enteric CH_4_ is produced during enteric fermentation in ruminants by anaerobic microorganisms collectively known as methanogens in the Archaea domain and the phylum *Euryarchaeota* [7]. The CH_4_ produced by animals is affected by many factors: the species, the production level and direction, the diet composition, the type of carbohydrates present, the level of ingestion, the degree of lipids saturation, some environmental factors such as temperature, and genetic factors such as feed conversion efficiency [8]. Searching for feasible mitigation strategies is essential to reduce CH_4_ emissions and increase feed efficiency. CH_4_ emissions from ruminants not only aggravate the global GHG effect but can also cause energy losses in livestock, accounting for 3.9–10.7% of ingested metabolic energy [9], representing one of the most important inefficiencies and economic losses in ruminant production systems [10].

Recognizing the importance of this issue, the FAO has been actively studying methane and mitigation strategies since 2005, when it began developing the Global Livestock Environmental Assessment Model (GLEAM). The first version was released in 2010, and GLEAM has since been continuously updated to enhance the accuracy of GHG emission estimates across species, regions, and production systems. In addition to the FAO, numerous researchers around the world are actively investigating innovative approaches to reduce methane emissions. Simultaneously, the Intergovernmental Panel on Climate Change (IPCC) has played a crucial role by providing global guidelines for estimating emissions. In 2006, the IPCC published the 2006 Guidelines for National Greenhouse Gas Inventories, which established standardized methods for calculating emissions from agriculture, including livestock. These guidelines are continuously updated, with the most recent update occurring in 2019, to incorporate advancements in estimating methane from enteric fermentation and other agricultural sources [3].

The data mining approach, also known as text mining (TM), is useful for extracting various information from large text databases [11]. Finding the most relevant words in a text and identifying significant patterns in text data are the aims of TM analysis. Text mining employs methods from computational statistics and machine learning [12]. Another type of text analysis is topic analysis (TA), which is a technique for identifying hidden textual patterns and the structure of significant themes within record collections using probabilistic models [12]. Quantitative analysis organizes knowledge on a specific subject/topic/discipline and identifies the main trends in a specific sector [13]. This is because the scientific literature contains potentially new knowledge [12]. Recently, in many sectors, there has been a growing interest in this kind of text analysis. This is largely attributable to the advancement of recent analysis techniques (e.g., using 4.3.1 R version, 3.7.0 Python version) and the availability of platforms that provide reference data (e.g., Scopus, Web of Science). Even in the livestock sector, several papers have recently been published on several topics, from precision livestock farming [13] and automated milking systems [14] to the well-being of several species, including beef cattle [15], horses [16], and buffaloes [17], utilizing techniques such as text mining and topic analysis.

Enteric CH_4_ emission is a hot topic in the livestock sector, but no TM and TA study has been conducted on it to date. For this reason, the goal of this review was to use TM and TA techniques to describe the literature’s evolution and geographical distribution, identifying the most investigated research topics and highlighting the knowledge gaps regarding enteric CH_4_ emission from ruminants.

## 2. Materials and Methods

### 2.1. Dataset

A bibliometric study of documents related to enteric methane emission (EME) in ruminants using a bibliometric database of Elsevier©, namely, Scopus^®^, was carried out. The topics were searched using keywords. Enteric methane (EM) AND another keyword such as “emission,” “ruminants,” “cow,” “buffalo,” “sheep OR goat,” “additive,” and “microbiome OR microbiota” were used in this research. The selected dates ranged from 1986 to May 2024. Several filters were added, including the selection of publications from scientific fields like agricultural and biological sciences, environmental sciences, biochemistry, genetics and molecular sciences, veterinary sciences, engineering, earth sciences, and planetary sciences. Under these circumstances, 3624 records were generated. Each search was exported as a Comma Separated Values file (Microsoft Excel^®^, v16.0, Redmond, WA, USA); then, the files were merged into a single file containing 3624 lines. Table 1 shows the total number of documents for each pair of keywords. The data were arranged in a tabular manner using the Excel spreadsheet (Microsoft Excel®, v16.0), with each record being shown in a row with its contents arranged in columns. The columns contained information about the title, author, year of publication, affiliation, type of record (e.g., article or review), source of publication (i.e., name of the journal), language, and abstract. Screenings were conducted on this single file to eliminate documents that were not in English, those in which the abstract was absent, those that misspelled “erratum,” and, finally, those in which the author was absent. After this, duplicate documents were eliminated. The articles dealing with rats, humans, manure, and soil were not included. In vitro studies were included due to their significant importance. 

**Figure 2:**
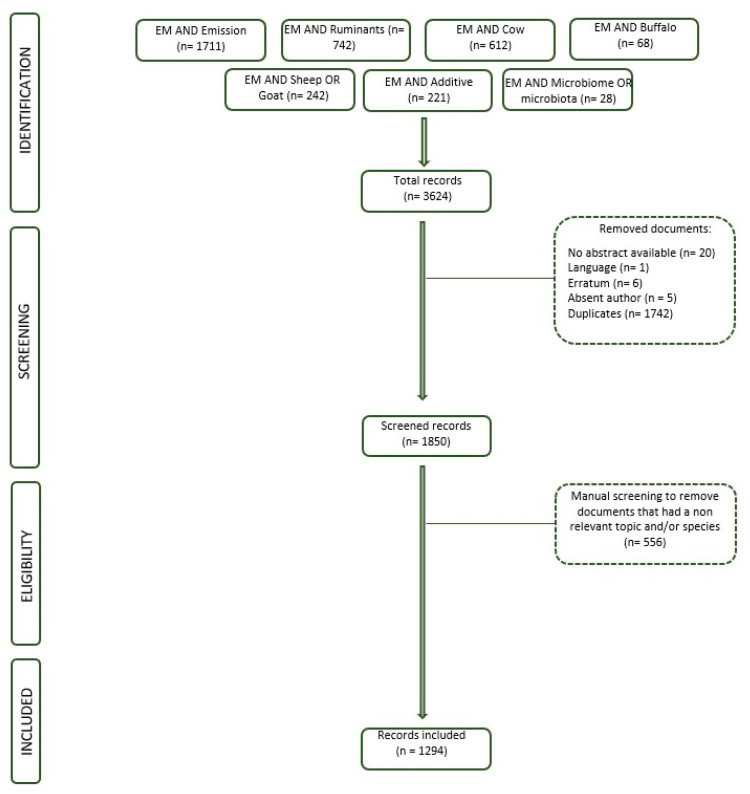
Flow chart illustrating the process of searching for and selecting scholarly literature on EME. The dashed lines indicate the quantity of excluded records and the rationale behind their removal from the study.

Finally, a manual screening of 1850 articles was performed. The screening process involved reading each abstract carefully to evaluate the relevance of the study to our review’s scope. The criteria for inclusion were as follows: (i) the article had to focus on enteric methane emission (EME) from ruminants, (ii) the study needed to present experimental or empirical data related to EME measurement, mitigation, or related environmental impacts or be a review of published data, and (iii) the article had to focus on ruminant species (cattle, sheep, goats, etc.). Articles were excluded if they did not focus on EME, were unrelated to methane emissions in the livestock sector, or addressed monogastric species (pigs, poultry) without reference to ruminants. Where abstracts did not provide enough detail, the full article was reviewed to ensure proper inclusion or exclusion. Details regarding the exact number of records downloaded from each research string, the eligibility procedure, and the initial screening are displayed in the flow diagram (Figure 2). 

A total of 1294 abstracts were selected, and descriptive statistics were performed on these articles regarding the year of publication, country, and journal of publication using Excel Pivot tables and graphics. Each record was associated with the nationality of the first author, and this information was displayed on a world map.

### 2.2. Text Mining

The TM technique transforms text into numerical data by emphasizing word frequency distributions, allowing for the identification of primary terms and their correlations within a data corpus. Text mining analysis was performed to identify the primary terms in the data corpus and their correlations. Text mining analysis was performed with the R studio environment [18] using a combination of functions in the package’s “tm” (v. 0.7.11) [19], “snowball” (v. 0.7.1) [20], “ggplot2” (v. 3.4.4) [21], “dplyr” (v. 1.1.4) [22], and “tidyverse” (v. 2.0.0) [23] (information about the packages used is provided in the Supplementary Materials).

The abstracts of the 1294 selected documents were compiled into an Excel file. This file contained two columns: one with the document’s progressive ID and another labeled “text”, which contained the abstracts. Text mining was then performed using this dataset. Before TM analysis, the three stages of pre-processing, tokenization, filtering, and stemming, were conducted [24]. Tokenization involves splitting text into individual words or phrases and converting these words to their base forms. This prevents counting the same word more than once when it appears in various grammatical forms [25]. Tokenization and filtering phases were conducted as follows:

- Convert text to lowercase;
- Remove strange symbols and punctuations (“@”, “/”, “*”);
- Remove numbers and extra white spaces;
- Remove common English language words such as articles, prepositions, and conjunctions (e.g., “the,” “a,” “and,” “on,” “at,” etc.) as they provide little information about the contents of the corpus;
- Remove stop words: “emission,” “enteric,” “methane,” “buffalo,” “cow,” “sheep,” “goat,” “ruminants,” “cattle,” “additive,” “microbiome,” “microbiota”.

In the final phase, text stemming was performed to reduce words to their root forms, thereby standardizing word representation and enhancing the accuracy of word frequency and association analysis. The words were then organized into a document-term matrix (DTM), with terms arranged in columns and documents in rows. The term frequency–inverse document frequency approach (TF-IDF) was used to give terms a relative weight [26]. This displays a term’s frequency adjusted for its total usage, highlighting the word’s significance across a collection of texts. The words with the greatest relevance (TF-IDF ≥ 9.5) were represented as a histogram, and a cloud of the most relevant words was created using the word clouds website (https://www.wordclouds.com/, accessed on 30 August 2024). Finally, the association between words with TF-IDF ≥ 9.5 was explored. The frequency of co-occurrence of word pairs was measured, considering a correlation of 1 when two words always appeared together. Associations with a coefficient of correlation greater than 0.40 were considered significant.

### 2.3. Topic Analysis

Topic analysis is a methodology used to uncover semantic connections hidden within documents. For the TA of our abstract corpus, we employed latent Dirichlet allocation (LDA), a widely recognized approach for topic modeling analysis [27]. Using a Bayesian probabilistic technique, words that frequently co-occurred in the documents to identify thematic areas were analyzed. The words used in the TA were drawn from the titles, keywords, and abstracts of the 1294 scientific literature records. The LDA function was used with the Gibbs sampling option of the “topic models” package in R [28]. Since the “optimal” number is typically unknown, multiple trials with varying numbers of topics were conducted (e.g., 5, 7, 9). The perplexity index and log-likelihood harmonic means were used to determine the appropriate number of topics. Nine topics were selected at the end of the procedure because they produced the most logical outcomes, with more than 5% of the papers in each topic. Each topic was represented as an individual bar histogram with the probability of the first 10 words inside each topic (based on beta values). Once the right number of topics was found, we proceeded with topic labeling based on the first 10 words and evaluated the types of articles that fell back into each topic. An Excel spreadsheet was used to provide descriptive statistics of the number of papers and the initial year of publication for each topic.

## 3. Results

### 3.1. Descriptive Statistics

**Figure 3:**
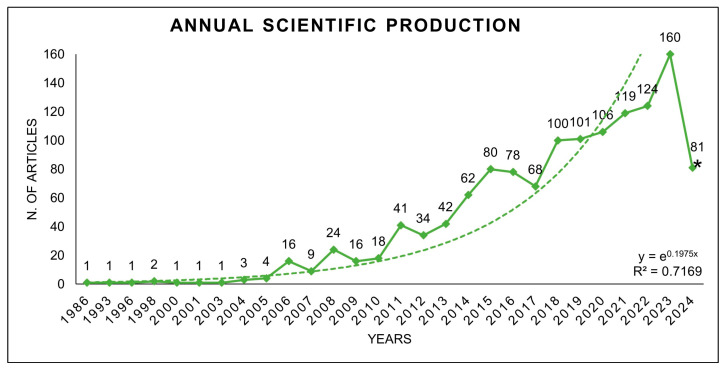
Records from 1986 to 2024 of peer-reviewed scientific literature (n = 1294) pertaining to EME in livestock sector. * The asterisk on the year 2024 indicates that results for that year are related to the period from January to May.

The distribution of the initial bibliographic search results by string on titles, abstracts, and keywords is reported in Table 1. Most articles were related to the string “enteric methane emission” (47%), followed by “enteric methane ruminants” (20%), “enteric methane cow” (17%), “enteric methane sheep and goat” (7%), “enteric methane additive” (6%), “enteric methane buffalo” (2%), and, lastly, “enteric methane microbiome or microbiota” (1%). After eliminating overlapping records and manually removing unnecessary ones, 1294 records were retained for further examination. The trend in the number of publications per year from 1986 to May 2024 is reported in Figure 3. It can be seen how publications on the subject began to grow from 2005 onwards, with an exponential increase (R^2^ = 0.72). The literature search was conducted in May 2024, so the number of publications related to this year is not complete.

**Figure 4:**
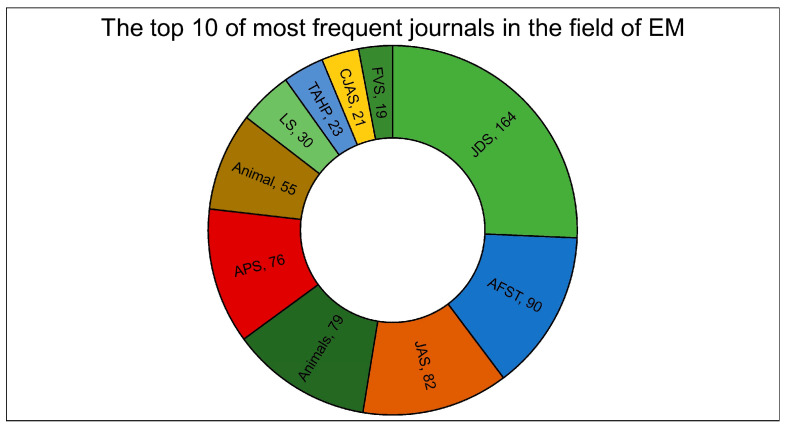
The top 10 most frequent journals in the field of EME from 1980 to 2024 with the relative number of articles. JDS: *Journal of Dairy Science*; AFST: *Animal Feed Science and Technology*; JAS: *Journal of Animal Science*; APS: *Animal Production Science*; LS: *Livestock Science*; TAHP: *Tropical Animal Health Production*; CJAS: *Canadian Journal of Animal Science*; FVS: *Frontiers in Veterinary Science*.

Figure 4 shows the first 10 journals in which articles relating to EME were published. In terms of percentages, the “*Journal of Dairy Science*, JDS” published 12.7% of the total articles, followed by “*Animal Feed Science and Technology*, AFST” (7%), “*Journal of Animal Science*, JAS” (6.3%), “*Animals*” (6.1%), “*Animal Production Science*, APS” (5.9%), “*Animal*” (4.3%), “*Livestock Science*, LS” (2.3%), “*Tropical Animal Health and Production*, TAHP” (1.8%), “*Canadian Journal of Animal Science*, CJAS” (1.6%), and, lastly, “*Frontiers in Veterinary Science*, FVS” (1.5%). Regarding the types of documents, the most published on the subject were original articles (82.1%), followed by reviews (10.5%) and conference papers (4.9%); the rest were book chapters and short surveys (2.5%). The most cited article (932 citations) was published in 2000 and concerned “Methane production by ruminants: Its contribution to global warming” [29]; this was followed by a review published by Beauchemin et al. [30] with 755 citations. The average number of citations of the 1294 documents was 24.9.

**Figure 5:**
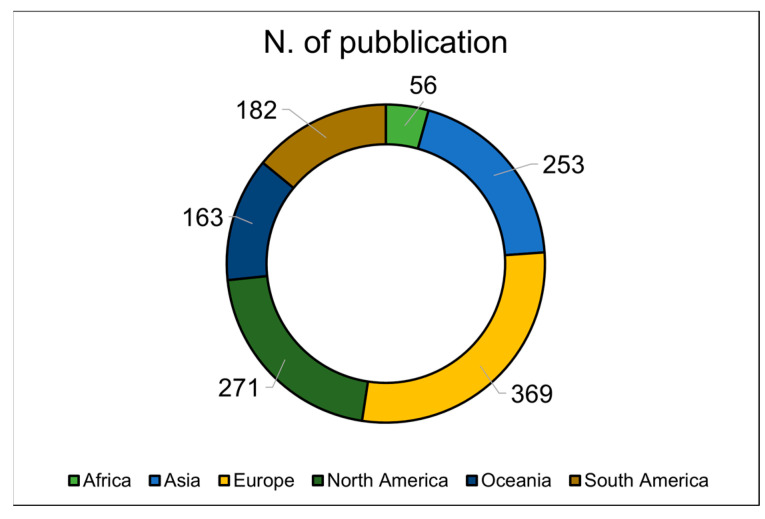
Pie graph depicting the number of scientific publications per continent.

Figure 5 illustrates how the 1294 scientific papers were distributed throughout the continent according to the first author’s affiliation country. The number (and percentage) of publications by continent was 369 (28.5%) from Europe, 271 (20.9%) from North America, 253 (19.6%) from Asia, 182 (14.1%) from South America, 163 (12.6%) from Oceania, and 56 (4.3%) from Africa.

**Figure 6:**
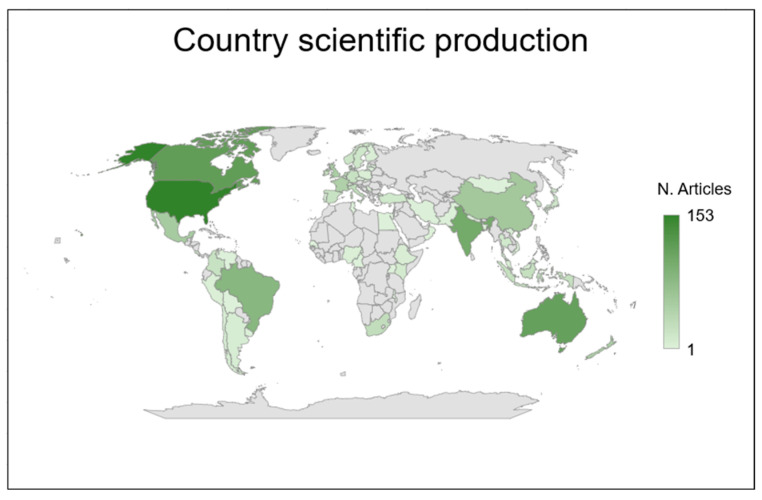
World map with the representation of the number of publications for each country (based on the nationality of the first author).

Figure 6 shows a geographical map indicating the number of publications per country (a darker color indicates a greater number of publications). The first countries with the highest number of publications were then explored for each continent. Concerning Europe, the countries with a greater number of publications were the United Kingdom (50), France (46), and the Netherlands (41). For North America, the first two were the United States (153) and Canada (117). India (101), China (57), and Indonesia (25) represented the top three states with the highest number of publications in Asia. For South America, the top three states were Brazil (82), Mexico (53), and Colombia (22). Australia (113) represented the state with the highest number of publications in Oceania, followed by New Zealand (50). Finally, as far as Africa was concerned, the greatest number of publications came from South Africa (25), followed by Kenya (10) and Ethiopia (4).

### 3.2. Text Mining 

**Figure 7:**
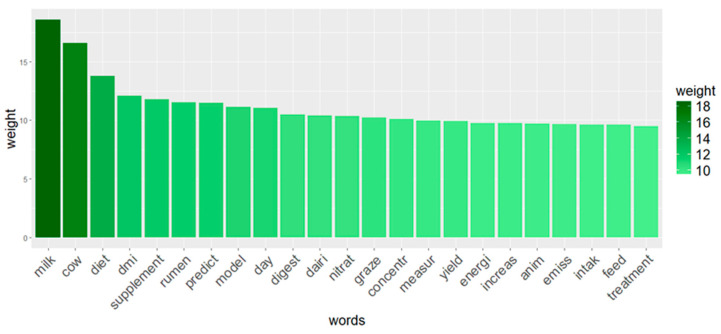
Histogram representation of the most relevant words (stems) in the database (TF-IDF ≥ 9.5).

**Figure 8:**
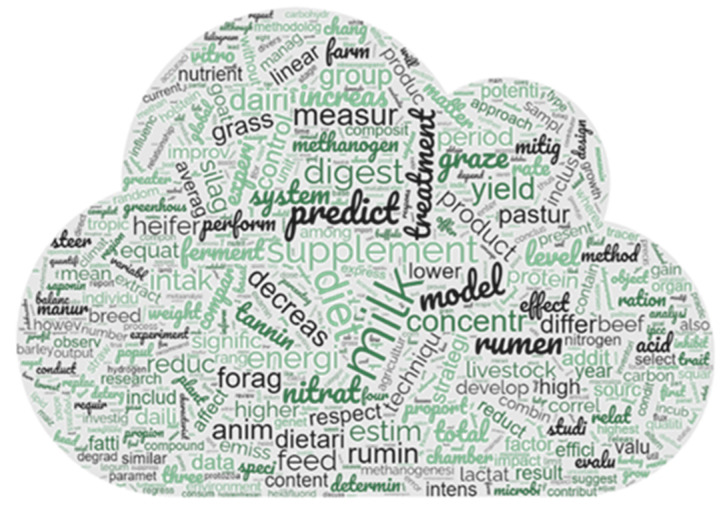
Word cloud of the most frequent words (TF-IDF values ≥ 9.5) of the 1294 records included in this study.

To determine which words in the data corpus were the most frequent, TM analysis was carried out. Following the data pre-processing, 1288 root terms were kept from the 1294 documents that were chosen after sparseness was reduced (i.e., “rare words” were excluded). “Rare words” were words that appeared only once or a few times in the entire corpus. These words were excluded during data pre-processing to avoid noise and improve the efficiency and effectiveness of the analysis. The results of the most frequent root words with a weight over 9.5 of TF-IDF are reported in Figure 7. The weights of the terms found (calculated as TF-IDF) ranged from 18.57 to 0.50. The word “milk” achieved the greatest value (TF-IDF of 18.57). The other words with the highest TF-IDF were “cow” (16.57), “diet” (13.76), “dmi” (12.10), “supplement” (11.81), “rumen” (11.54), “predict” (11.48), “model” (11.16), “day” (11.07), “digest” (10.49), “dairi” (10.43), “nitrat ” (10.35), “graze” (10.25), “concentr” (10.09), “measur” (9.97), “yield” (9.95), “energi” (9.76), “increas” (9.75), “anim” (9.72), “emiss” (9.67), “intak” (9.64), “feed” (9.63), and “treatment” (9.51). A word cloud with the most frequent terms is shown in Figure 8, in which the size of the font is proportional to the TF-IDF of each term. Table 2 shows the correlations between the most relevant terms (r ≥ 0.40) and the other terms in the matrix. The terms “anim,” “concentr,” “dairi,” “day,” “diet,” “dmi,” “emiss,” “feed,” “intak,” “milk,” “nitrat,” “rumen,” “supplement,” and “yield” did not show any correlation.

### 3.3. Topic Analysis

**Figure 9:**
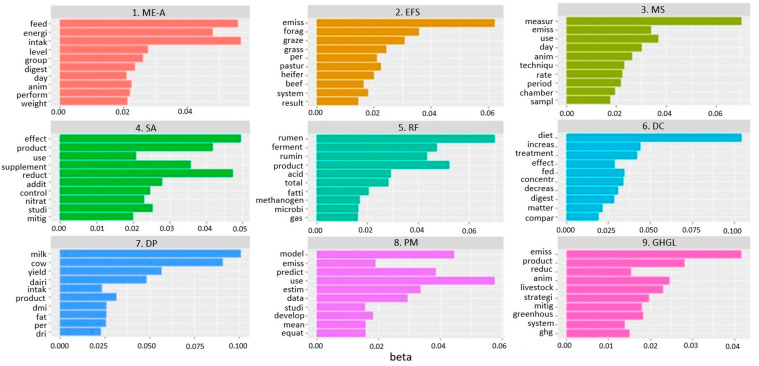
Histograms showing the most relevant terms for each of the 9 topics in the latent Dirichlet allocation (LDA). The top 10 terms with the highest beta values are shown. beta = probability that a word corresponds to a certain topic.

The first 10 words of the nine topics are displayed in Figure 9. Table 3 shows a list of the nine topics, their nomenclature, the number of articles published (and relative percentage), and the year of first publication for each topic. The topic that showed the most documents was topic 9, “Greenhouse gas emission from livestock” (18.08%), followed by topic 6, “Diet composition” (13.45%); topic 8, “Prediction model” (11.28%); topic 4, “Supplement and additive” (10.97%); topic 5, “Ruminal fermentation” (10.20%); topic 3, “In vivo measurement system” (9.81%); topic 2, “Extensive farming system” (9.27%); topic 7, “Dairy production” (9.20%); and, finally, the topic that showed the lowest percentage of published documents was topic 1, “Methane emission-animal” (7.73%).

## 4. Discussion

This study aimed to evaluate the evolution of EME in the livestock sector through a machine learning approach with TM and TA analysis methods from 1986 to May 2024. The number of publications relating to EME in the livestock sector is growing. The first published article was from Germany, dated 1986, and published by [31], who aimed to provide an overview of the global CH_4_ production by people, domestic ruminants, and wild ruminants. Those authors concluded that CH_4_ production by domestic and wild animals contributed to about 15–25% of the total tropospheric CH_4_. Until 2004, publications were fluctuating and limited. Starting from 2005, there was an increase in publications until 2019, in which the number of publications exceeded 100 articles/year. The rising number of publications on EME reflects the growing interest in this potent GHG due to its significant climate-altering effects, with a notable increase in attention since 2005. That year saw the entry into force of the Kyoto Protocol signed in 1997 by approximately 40 developed countries. The protocol sets binding targets for reducing GHGs for industrialized countries, also referred to as developed countries. The main objective of the Kyoto Protocol was to limit the increase in the global average temperature to less than 2 °C above pre-industrial levels. Furthermore, the Kyoto Protocol paved the way for international negotiations and agreements on climate change, including the annual Conference of the Parties (COP) under the United Nations Framework Convention on Climate Change (UNFCCC). The COP serves as the main decision-making body for global climate policy, where countries come together to negotiate and make decisions and agreements to address climate change. Another significant milestone in efforts to curb GHG emissions was the 2015 Paris Agreement. This agreement outlined the goal of limiting the increase in the global average temperature to well below 2 °C and aiming to keep it within 1.5 °C. The heightened focus of the scientific community on addressing climate change and reducing GHG emissions, particularly from EME, is evident in the surge of publications following 2005, with a peak coinciding with the adoption of the Paris Agreement in 2015. 

The largest number of publications was produced by the *Journal of Dairy Science* (JDS), followed by *Animal Feed Science and Technology* (AFST), *Journal of Animal Science* (JAS), and *Animals*. These journals’ research scopes are in the livestock sector, in particular relating to dairy science (in the case of JDS); feeds, as in the case of AFST; and livestock much more generally in the case of the last two journals (JAS and *Animals*). These journals reflected the most common terms discovered with TM, namely, “milk”, “cow”, and “diet”, which will be discussed later.

The geographical distribution of publications based on the first author showed that the countries with the highest number of published articles corresponded to the Northern Hemisphere, where there is the greatest production of anthropogenic GHGs [32]. Additionally, in these areas, there is a high number of farms, and there is a greater contribution of funding for research in the livestock sector [32]. According to the FAO [3], regions with high milk production include Europe, South Asia, and North America, whereas meat production is concentrated in East Asia, Europe, and North and South America. According to findings reported by [32], studies on EME measurement techniques were conducted predominantly in Europe (32%), followed by Oceania (23%), North America (20%), Asia (15%), South America (9%), and Africa (1%). Additionally, Della Rosa et al. [32] highlighted that most studies focused on cattle (64%) and sheep (22%), with only 7% involving goats, 5% involving buffalo, and 2% involving other ruminants such as alpaca, bison, llama, and deer (Table 4). Their review findings partially align with our own, indicating a similar distribution of focus in terms of the geographical regions studied.

### 4.1. Text Mining

From TM analysis, the most frequent words were “milk”, followed by “cow”, “diet”, and “dmi”, with TF-IDF higher than 12. It is not surprising that the word “milk” followed by “cow” were the most common ones in our survey on EME in the livestock sector. Looking at the world map of the countries in which most of the articles were published, the same countries are the largest breeders and producers of cow milk, according to FAO data [3]. The word “cow” is directly linked to EME as, among all the livestock species, cattle represent the largest producers of GHGs (62%). As discussed before, the major component of CH_4_ is given by enteric fermentation compared with monogastric animals. Methane production can be expressed in three different ways: (i) CH_4_ production in liters or grams per day; (ii) CH_4_ yield, defined as liters or grams of CH_4_ per kilogram of dry matter intake (DMI); and (iii) CH_4_ intensity, defined as liters or grams of CH_4_ per kilogram of milk/meat produced [33]. Therefore, the word “milk” could be related to the expression of the intensity of EME.

The word “diet” represents the rations provided to animals. Diet plays an important role in modulating the EME in ruminants. Indeed, among the top important terms highlighted by the TM, terms related to nutrition emerged, such as “diet”, “supplement”, “digest”, “graze”, and “concentr”. Several studies agreed that diet modulation remains the most straightforward and inexpensive approach to lessen EME [34,35,36]. Methane output could be lowered by 10% to 40%, depending on the type of feeding strategy intervention [37]. Numerous feeding strategies for mitigating CH_4_ are currently in use. These strategies can be summarized in three broad categories (adapted to [34]): (i) dietary supplementation of feed additives that either directly block methanogens or change the metabolic pathways, resulting in the reduction of the substrate for methanogenesis; (ii) improving the quality of the forage and modifying the forage–concentrate ratio; and (iii) improving feeding management. The word “dmi” (DMI—dry matter intake) is a very important acronym in the context of EME since there is a strong correlation between DMI and EME [9,38,39,40]. Notably, multiple EME prediction models are based on DMI. Some studies [41,42] demonstrated that DMI predicts CH_4_ production (g/d), with a coefficient of determination (R^2^) of 0.60 and 0.64, respectively. Furthermore, as previously discussed, DMI is used as an expression of CH_4_ yield (g of CH_4_/kg of DMI). It has been observed that this is a precise method to evaluate how effective a mitigation strategy is, regardless of possible changes in feed intake, given that feed intake is the main factor driving CH_4_ production [36]. To confirm the above, following the word “dmi”, “predict” and “model” were found. Even though the remaining words had a lower weight according to the TF-IDF analysis (<9.5), they were all noteworthy. By studying key factors such as cow diet, DMI, supplement usage, and grazing management, it is possible to identify effective strategies to reduce GHGs without compromising milk production or cattle health. Utilizing mathematical models and predictive methods allows one to anticipate the impact of these strategies and develop tailored solutions to mitigate increases of CH_4_ emissions. Furthermore, improving the energy efficiency of cattle metabolism can decrease overall GHGs from the dairy and meat industries.

### 4.2. Topic Analysis

From the TA, several expected topics emerged, ranging from ruminal fermentation (RF) to CH_4_ measurement systems (MS), diet composition (DC), and topics representing extensive farming systems (EFS). We were also expecting a topic for meat production (just like the one that emerged for dairy production), but this could have been hidden in the other topics. For example, the term “beef” was found in topic 2. The topic that generically encompassed the subjects dealing with GHGs was the one that emerged before the others (1986) and the one with the highest number of articles published (18.08%) in the present survey. Topic 9 was generically defined as “greenhouse gas from livestock, GHGL”, and the most important terms that emerged from the TA reflected this topic (“livestock,” “strategi,” “greenhouse,” “ghg,” “mitig”). Furthermore, compared with the other eight, in this topic, there was a large majority of reviews (32% of documents), probably because it was very generic. This broad topic included studies that, indirectly, quantified EME, and had, as their general objective, an estimation of the life cycle assessment (LCA) of livestock supply chains. Initially, the studies on LCA focused on the primary sector, while only a few included additional post-agricultural components (e.g., transport) in the analysis [43]. Life cycle assessments of several farming systems showed that on-farm emissions are the largest contributor to the carbon footprint of dairy or beef supply chains [43,44,45]. The carbon footprint, an indicator calculated with an LCA approach, evaluates the GHGs associated with the life cycle of a product [44]. A cradle-to-grave LCA study on organically farmed beef [44] reported that the greatest production of GHGs (75–89%) along the food chain is associated with farms and that EME was the greatest source of GHG arising directly from agricultural activities (47%), with certain variability due to the different types of farming systems (conventional, organic) and/or methodological approaches adopted. Thoma et al. [43] reported that even if a substantial majority of GHGs are derived from enteric CH_4_, the impacts of the entire chain and supply chain can be reduced.

Topic 2 contained terms related to extensive farming systems (e.g., “graze,” “forag,” “pasture,” and “grass”); hence, it was defined as “extensive farming system, EFS”. This topic encompassed more than 9% of the publications considered in this review, representing the fifth most important topic identified in the TA. Extensive farming systems likely warranted a separate topic because they exhibit distinct characteristics that differentiate them from other farming systems. Systems based on pasture constitute a substantial contributor to GHG. Although grazing systems are widely utilized, there are difficulties in measuring and reducing the CH_4_ emissions from these systems [46]. Furthermore, grazing systems are one of the most significant habitats for CH_4_ exchange. Their CH_4_ budget comprises two main sources: soil bacterial populations that, depending on the soil’s physical and biological conditions, can either produce or consume CH_4_, and ruminants on pasture that generate CH_4_ during the digestion of grass. Such systems are also considered inefficient and associated with low animal performance, tending to increase the emission rate per unit of products (CH_4_/kg of milk/meat) [47,48]. These issues are more pronounced in developing and poor countries, such as African countries, which currently stand as some of the largest GHG emitters [48]. In these regions, pasture is scarce, and what is available is often of poor quality, resulting in digestive inefficiency and, consequently, increased emissions [49]. On the other hand, EFS is considered a potential GHG mitigation practice [50]. Although extensive systems are recognized as carbon sinks, and silvopastoral systems can be effective in protecting animals from extreme weather conditions [51], it has been observed that proper pasture management (e.g., adjusted grazing intensity, fire management, legume or grass sowing, pasture fertilization) leads to soil carbon sequestration, averaging 1.76 t CO_2_ ha^−1^ per year [52]. Agro-ecological factors, as well as historical and current farming practices, significantly impact soil carbon sequestration rates [53]. The complexity of interactions between soils, vegetation, grazing animals, and human interventions makes it challenging to categorize them in farming management categories typically evaluated in the scientific literature; thus, assessing the sequestration potential of grazing practices remains one of the major challenges [53].

Topic 3 was one of the expected ones. Among the most important terms, the terms “measur”, “animal”, “techniqu”, and “chamber” fell entirely within “in vivo measurement systems, MS”. More than 9% of the selected articles were included in this topic. The trend of publications has experienced some fluctuations over the years. The variations in the number of publications over time could be linked to the introduction of new measurement systems. For example, in 2010, the commercial GreenFeed (GF) system (C-Lock Inc., Rapid City, SD, USA) was introduced. Since 2015, studies utilizing the GF system have been conducted, but its adoption began around 2016, evidenced by a peak in publications during that year. In 2016, within topic 3, over 45% of the studies were conducted using the GF system. It is obvious that after the introduction of a new measurement system, many studies seem to verify the actual efficiency of the instrument. Several techniques have been developed to measure CH_4_ emissions from individual animals, despite the UNFCCC (1997) stating that “comparable methodologies” should be used to compile a GHG inventory to make national results comparable in a consistent manner [32]. Every technique has an impact on the variability of EME, and different methodologies may add unpredictability to national inventories and assessments of CH_4_ emissions [54,55].

To date, several methods are available to measure CH_4_ emissions from ruminants [56,57]. None of them is ideal, and each has pros and cons. The best approach will rely on the goal, tools, expertise, time, and resources available to help manufacturers and researchers develop and track effective CH_4_ mitigation techniques [56,57,58]. Although the number of techniques available for EME measurements has increased during the last decade, respiration chambers (RC), sulfur hexafluoride (SF6) tracers, and GF automated emissions monitoring systems are the most used. Della Rosa et al. [55] reviewed 397 studies published between 1995 and 2018 and reported that the majority of EME measurements were performed with RC (55%), followed by SF6 (38%) and, lastly, GF (7%). The remaining 6%, mostly from Europe, comprised 2% face mask (FM), 2% sniffer (SNF), 1% laser methane detector (LMD), and 1% portable accumulation chamber (PAC) (from Oceania) methods.

There is uncertainty in all measurement techniques due to random components such as changes in animal diets, management practices, and environmental conditions. Current methods still have the potential to over- or under-estimate the reference level of EME in ruminants due to their random factors [58]. Furthermore, a source of variability is attributed to different measurement procedures, which could be reduced using similar settings and protocols within each technique [55]. The measurement of EME directly on the animal, although with varying degrees of accuracy, is an expensive and burdensome method [33,59] and, therefore, it is not feasible for large-scale routine measurements, an essential requirement for genetic selection [33]. Given the need for cheaper, more rapid methods to be used on a large scale, empirical methods for estimating EME have become widespread [60]. Therefore, identifying proxies (e.g., indirect indicators or traits) that are related to CH_4_ emissions, but that are easy and relatively cheap to record on a large scale, are a much-needed alternative [33].

In topic 8, terms such as “model”, “predict”, “data”, “equat”, and “estim” were highlighted, all terms related to EME estimation models using proxies. For this reason, this topic was called “Prediction model, PM”. Since CH_4_ is the result of feed rumen fermentation (the production of CH_4_ is related to feed intake and fermentability; [42,47]), most predictive equations are based on DMI or metabolizable energy (ME) and gross energy (GE) intakes and the amount of NDF/ADF ingested [42]. Several milk fatty acids (MFA) have been suggested as potentially useful for EME prediction [61,62,63]. However, predictive models based on MFA were developed using a narrow range of diets and limited data [64]. Milk fatty acids show a potentially effective proxy since fatty acids have common biochemical pathways in the rumen with CH_4_ [64]. In particular, odd-chain and branched-chain fatty acids have a strong relationship with the molar proportions of individual volatile fatty acids (VFAs) in the rumen [61,63]. The fatty acids with greater proxy activity are C14:0 iso, C15:0 iso, and C17:0, which are positively related to CH_4_ production [61,62]. Recently, in a meta-analysis, Bougouin et al. [64] observed that the prediction equations considering only MFA showed a higher mean square error in estimating CH_4_ emissions expressed as production, yield, and intensity (65.1 g/day, 2.8 g/kg of DMI, and 2.9 g/kg of milk, respectively) compared with more complex equations. The complex equations included DMI, NDF, ethereal extract, days in milk, and body weight, for which the mean square error in EME estimation was reduced (46.6 g/day, 2.6 g/kg of DMI, and 2.7 g/kg of milk, respectively, for production, yield, and CH_4_ intensity). Jonker et al. [65] reported that DMI has an effect of over 90% in the prediction of CH_4_ emissions.

Rumen size and feed retention time, according to Goopy et al. [66], are factors that affect the CH_4_ yield as more rapid passage of the material from the rumen translates into a reduction in the time available to ferment the substrate. It was observed by Nkrumah et al. [67] that the duration of feeding and the presence of animals in the feeder were related to CH_4_ production, suggesting the influence of feeding behavior on circadian patterns of CH_4_ production [68]. Other authors [69] studied rumination time in dairy cows as a possible proxy for EME and observed that cows with high rumination activity produced more milk, consumed more concentrate, and produced more CH_4_ than cows with low rumination time. Identifying easily measurable proxies would allow for large-scale data as with the advent of precision livestock farming it is now possible to physiologically monitor (feeding time, rumination time, etc.) various parameters 24 h a day [70].

Topic 5 was defined as “Ruminal Fermentation, RF” because, looking at the words highlighted by the beta probability of the LDA analysis, the most important words were “rumen” followed, in order of importance, by “ferment,” “rumin,” “acid”, etc., all words associated with RF. This topic encompassed numerous articles focused on in vitro trials. Such trials play a pivotal role in studying EME by offering a controlled, efficient, and targeted experimental approach to comprehensively understand and address EME.

The majority of methanogenesis takes place in the rumen, and a lesser proportion (~13%) of CH_4_ is produced in the cecum and colon [71]. The microorganisms present in the rumen degrade the feed, producing short-chain fatty acids (acetic, butyric, and propionic acid), CO_2_, and metabolic hydrogen (H_2_). Short-chain fatty acids (SCFAs) are absorbed through the rumen wall to provide energy. Methanogens, belonging to the Archaea domain, utilize H_2_ and CO_2_ to produce CH_4_ [72]. In this process, CO_2_ is the carbon source and H_2_ is the main electron donor. Four moles of H_2_ can produce one mole of CH_4_ [73]. Methanogenesis is the main biochemical pathway to remove metabolic H_2_ to maintain a very low concentration of H_2_ in the rumen and ensure proper feed digestion [29]. The concentration of H_2_ affects the feed degradation rate. If the concentration of H_2_ increases, the rate of feed degradation decreases. Methane emissions can be reduced by inhibiting H_2_ formation from fermentation or promoting alternative H_2_ pathways [73]. While methanogenesis is predominantly driven by methanogenic bacteria, the correlation between EME and the concentration of these bacteria appears ambiguous in several studies [33]. Some investigations have uncovered significant positive relationships; others have found no correlation between the concentration of methanogens and methanogenesis [74]. Additionally, Bouchard et al. [75] reported a reduction in methanogens without a significant decrease in CH_4_ production. Methanogens are the dominant organisms in the rumen; there are 10^6^–10^8^ cells/mL of methanogenic Archaea per ml of rumen fluid [76]. Other microorganisms, such as protozoa, indirectly influence CH_4_ emissions by using substrates (starch, cellulose, hemicellulose, pectin, and soluble sugar) to produce SCFAs and H_2_, which are then converted into CH_4_ by methanogens [77].

Methanogens are anaerobic organisms that thrive in environments with an optimal pH ranging from 6.0 to 7.5 [73] and are known to be sensitive to low pH. Van Kessel et al. [78], using ruminal fluid from fistulated cows fed two types of diets (high-forage or high-concentrate), demonstrated that methanogenesis is pH-dependent, with complete inhibition of CH_4_ production at pH values below 6.0, and highlighted that volatile fatty acids, such as acetic acid, can directly affect the activity of methanogens at low pH levels. Despite this sensitivity to low pH, methanogens may survive episodes of low ruminal pH through changes in community structure or sequestration in protected microenvironments within biofilms or protozoa [79], allowing them to persist and continue CH_4_ production under otherwise inhibitory conditions. Although ruminal methanogens are inhibited by a pH below 6.0 in vitro [78], CH_4_ production rates in vivo do not decrease when ruminal pH drops to levels associated with subacute or acute ruminal acidosis (5.2–5.5) in beef cattle [79]. This suggests that there are additional factors, such as increased propionate formation or passage rate, that contribute to lower EME in cattle fed high-grain diets compared to high-forage diets. Therefore, reducing ruminal pH alone is not considered an effective CH_4_ mitigation strategy [30]. 

Diet is a pivotal factor in EME studies; indeed, one of the emergent topics (topic 6) was precisely defined as “diet composition, DC”. The keywords that defined this topic were “diet”, “fed”, “concentr”, “digest”, and “matter”. This topic represented the second most important in the TA (with a percentage of articles exceeding 13%). Manipulating the diet has the potential to reduce EME and consequently increase the feed efficiency of animals, as CH_4_ production results in a loss of GE intake [80]. Some studies [81,82] demonstrated that the basal diet has significant effects on EME. Additionally, as will be discussed later, the composition of the basal diet has the potential to enhance the efficiency of certain supplements in reducing EME, showing additive effects [83,84]. The term “concentr”, which is the root of concentrate, emerged as a decidedly important term in this topic. Increasing the proportion of concentrates is associated with increases in the starch content in the diet. 

The ability of starch to reduce CH_4_ emissions has long been recognized. Starch fermentation produces propionic acid, providing an alternative H₂ sink to methanogenesis. Additionally, starch fermentation can lower ruminal pH, which can reduce the populations of protozoa and methanogens [81]. However, starch is rapidly fermented in the rumen, leading to high concentrations of VFAs and/or lactate, which increase the risk of acute or sub-acute ruminal acidosis, so this is proven to be an unsustainable method in attempting to reduce EME in ruminants [34]. The type of cereal and its processing technique can affect the fermentation level and EME. In terms of cereal sources, both absolute CH_4_ production and CH_4_ yield appear to follow the order of wheat and maize flakes < maize < barley, with the ranking strongly dependent on the composition and extent of grain processing [85]. Processing methods of cereals (applying different combinations of heat, moisture, time, and mechanical actions) can alter starch fermentation and ruminal pH. Hales et al. [86] observed a 17% reduction in CH_4_ yield with a steam-flaked maize-based diet compared to a dry-rolled maize-based diet in beef cattle. Herrera-Saldana et al. [87] showed that increasing the processing degree (i.e., smaller particle sizes) of cereal grains increased the rate of ruminal degradation of the grain and decreased ruminal pH. The inclusion of concentrated feeds in the diet of ruminants (>40% of the diet) has the potential to reduce CH_4_ intensity [47].

Forages represent a very important share in ruminant feeding, and their quality has been shown to have a high potential for modulating CH_4_ emissions [35]. Different types of forage can influence CH_4_ emissions due to differences in their chemical composition. High-quality forage, such as young plants, has more readily fermentable carbohydrates and less NDF, which increases digestibility and transit rate and can modify the fermentation pathway and minimize the formation of CH_4_ [34,88]. In contrast, more mature forages result in a greater loss of CH_4_, primarily due to an increase in the C:N ratio, which reduces digestibility [89]. The improvement of organic matter digestibility (%) and the increase in protein content (g/kg DM) of grass silage was negatively associated with CH_4_ yield (R^2^ = 0.74 and R^2^ = 0.36, respectively). In contrast, the NDF content (g/kg DM) showed a positive relationship with CH_4_ yield both in grass silage (R^2^ = 0.44) and in maize silage (R^2^ = 0.60) diets [35]. 

Applying current precision feeding techniques, it is possible to know the chemical composition of the raw materials and total mixed rations in real time [90]. This could be a rapid and advantageous method for predicting CH_4_ yield. Furthermore, it has been shown that forage particle size can affect CH_4_ emissions per kg of DMI. For example, chopping or pelleting forage can reduce EME per kg of DMI, as smaller particles are less degraded in the rumen [91]. The meta-analysis by Arndt et al. [92] reported that mitigation strategies such as decreasing grass maturity and reducing the dietary forage-to-concentrate ratio could decrease CH_4_ intensity by 12% on average (range 9 to 17%).

Dietary lipid supplementation lowers CH_4_ emissions. This could include lower levels of methanogen activity, smaller populations of protozoa, and less fermentation of ruminal organic matter [80]. Eugène et al. [93] in their meta-analysis reported that adding lipids to dairy cows’ diet decreased CH_4_ production by 0.305 g/kg of DMI for every 1% increase in ether extract, and this was primarily due to the reduced DMI. Furthermore, unsaturated fatty acid-rich lipids were subjected to biohydrogenation in the rumen, lowering the amount of hydrogen available for the generation of methane [30].

It is generally recommended not to exceed 6–7% of lipids on a DM basis in the diet, since greater levels can cause a depression in DMI, thus negating the benefits derived from the increased energy density of the diet [94].

Della Rosa et al. [32] reported that in most studies on EME measurement systems, 72% tested supplements and/or additives. Topic 4 (“supplement and additive, SA”) was characterized by the terms “supplement”, “addit”, “nitrat”, and “reduc”. Supplements or additives include inhibitors that are added to an animal’s diet with the aim of reducing CH_4_ formation. They act through different mechanisms [10,95]: (i) inhibition of CH_4_ production by reacting with methyl-coenzyme M, which is involved in the last step of methane formation; (ii) alternative H_2_ sinks that move hydrogen ions away from methanogenesis; (iii) inhibition of methanogenic Archaea bacteria and reduction of the number of protozoa; (iv) improved nitrogen metabolism; and (v) reduction in pH, increase in propionate, and decrease in the acetate/propionate ratio. A variety of additives, including ionophores, essential oils, nitrates, algae, and yeasts, were examined. Although many strategies have been proposed for mitigating CH_4_, many others are in the very early stages of development (e.g., phages, bacteriocins), have low mitigation potential (e.g., yeast, directly fed bacterial microbes, saponins, ionophores), or are difficult to apply on-farm (e.g., protozoan defaunation) [96].

According to Arndt et al. [92], sustainable strategies for enteric CH_4_ mitigation should preferably avoid socioeconomic and environmental trade-offs and, ideally, increase production yield per unit of input. This suggests that other than the reduction of enteric CH_4_ in absolute terms (g of CH_4_/day), an additive must be evaluated for its effectiveness in modifying animal performance, which, therefore, influences the yield of CH_4_. When choosing the feed additive, in addition to effectiveness and efficiency, the toxicity and potential environmental impacts/undesirable side effects must be considered [10]. Arndt et al. [92], in a meta-analysis carried out on 480 studies considering 98 mitigation strategies, reported that the most effective mitigation strategies in the context of “supplements” were CH_4_ inhibitors, tanniferous fodder, electron sinks, oils and fats, and oilseeds. These strategies decreased CH_4_ intensity by 17% on average (ranging from 12 to 32%) and daily CH_4_ emissions by 21% on average (ranging from 12 to 35%), without negatively affecting animal performance. Among the CH_4_ inhibitors, 3-nitrooxypropanol (3-NOP) is the one that has shown the greatest efficiency in CH_4_ abatement and the minimum effect on animal performance [92,97]. These compounds are specific inhibitors of methyl-coenzyme M reductase, an enzyme involved in the CH_4_ formation of methanogenic Archaea in the terminal phase [98].

The reduction of CH_4_ from 3-NOP varies with dosage [99,100] and depends on the type of diet [82,97,99]. Increasing levels of 3-NOP in the diet decreased enteric CH_4_ emissions per unit of body weight, CH_4_/kg DMI, CH_4_/L of milk, and CH_4_/kg of digested organic matter [82,101]. van Gastelen et al. [82] reported a greater reduction in CH_4_ intensity and yield with diets containing a higher starch content (based on corn silage) when supplemented with 3-NOP compared to diets based on grass silage.

The long-term effects of 3-NOP administration in dairy cows have been evaluated. In their year-long study covering all lactation phases, van Gastelen et al. [97] reported that 3-NOP persistently decreased CH_4_ emissions, with a positive impact on milk fat and protein yield, energy-corrected milk yield, and feed efficiency. 3-NOP is currently a commercial product available to farmers. Indeed, in 2022, the DSM company received EU market approval for Bovaer^®^ for dairy cows, following a positive EFSA opinion confirming that the product reduces enteric CH_4_ emissions from dairy cows and is safe for the animal and the consumer. This was the first time the EU marketed a product as a feed additive for its environmental benefits [102].

Topic 1 stood out from the others due to the presence of terms referring to animals like “intake,” “animal,” “performance,” “weight,” and “group”, so it was defined as a topic inherent to the interaction between EME and animal; for this reason, it was called “methane emission-animal, ME-A”. Approximately 8% of the articles selected fell into this topic. Generally, CH_4_ yield was reported to be affected by the level of intake, diet quality, supplementation level [103], physiological state (lactating, non-lactating) [104,105], and cattle class (beef, dairy) [81,106]. It has been suggested that CH_4_ emissions vary during different physiological phases [105,107]. For example, CH_4_ levels have been reported to increase by up to 35% from early to late lactation [38,108], and this increase was primarily due to an increase in DMI, the main driver of CH_4_ production. Lyons et al. [107] measured CH_4_ emissions in dairy cows at different times of lactation and reported an increase in CH_4_ yield (L/kg DMI) from the 5th to the 42nd week of lactation (32.2 vs. 36.7 L of CH_4_/kg of DMI, respectively). Furthermore, it has been observed that the lactation phase affects the level of reduction of EME by supplements. Adding 3-NOP to the rations of dairy cows, van Gastelen et al. [97] observed a reduction, on average, of 16%, 20%, 16%, and 26% in the CH_4_ yield (g/kg DMI) for the dry, early, mid, and late lactation diets, respectively. This variability was partly attributed to the different compositions of the diets for the different physiological phases and, more specifically, to the fiber content.

Oddy et al. [109] carried out a study on dairy sheep and reported that the main determining factor of CH_4_ production and yield was DMI. However, they also identified factors such as age, rumen volume, and pregnancy status as influential.

Dong et al. [110] further elucidated that the increase in EME with increasing age is related to greater DMI. In contrast, Ramírez-Restrepo et al. [111] reported similar average CH_4_ yields between heifers and multiparous cows, both estimated with the SF6 technique (25.3 ± 0.52 and 24.1 ± 0.55 CH_4_/kg DMI for heifers and multiparous cows, respectively) and measured in the RC (23.7 ± 0.66 and 23.6 ± 0.66 CH_4_/kg DMI for heifers and multiparous cows, respectively), suggesting that there were no differences based on age. In a recent study on grazing dairy cows, Salas-Riega et al. [112] reported that dry cows produced less CH_4_ than lactating cows (266 and 325 g CH_4_/cow/day for dry and lactating, respectively). Body weight (BW) also affects CH_4_ production. The effect of BW can be attributed to the relationship between BW and intestinal capacity, since intestinal volume is proportional to BW, thereby influencing DMI [113]. Animals with a higher BW typically have a greater intestinal volume, enabling them to consume more dry matter. This increased DMI is correlated with higher CH_4_ production.

Topic 7 was the topic linked to dairy production. The terms that emerged for topic 7 were “milk,” “cow,” “yield,” “dairi,” and “product” and, therefore, it was called “dairy production, DP”. This topic included studies conducted on dairy ruminants, particularly dairy cattle. Most studies on EME were conducted on dairy cows, as also confirmed by the meta-analysis by Della Rosa et al. [32]. Those authors reported that 62% of studies conducted on *Bos taurus* were on dairy cattle (4% growing, 50% lactating, and 8% non-lactating) and 38% involved beef cattle (35% growing and 3% mature). The terms found in this topic have been discussed extensively previously.

## 5. Conclusions

The aim of this review was to analyze the literature referring to EME in the livestock sector from 1986 to May 2024 using machine learning methods. A growing trend was observed in the number of publications relating to EME, especially in response to the global political interest in reducing global GHG emissions. At a geographical level, the publications reflect the major GHG-emitting countries, i.e., those in the Northern Hemisphere. From the text mining results, terms strongly associated with EME emerged, covering various aspects such as milk, cow, diet, DMI, supplement, model, measure, and animal. The results of the topic analysis highlighted expected topics such as GHGs from livestock, dairy production, diet composition, and ruminal fermentation, as well as unexpected topics like extensive livestock farming. Other subjects, such as beef production or genetic improvement, were evidently overshadowed by other themes. 

### Future Prospects

The future of EME research in the livestock sector is promising, with several key areas for development. Research should continue across multiple fronts, emphasizing the need for stronger connections between them. Increased funding from governments, all aiming to identify effective strategies for reducing EME, presents significant opportunities for advancement in this field. 

First, diet optimization remains critical, focusing on innovative feed additives to reduce EME while maintaining animal health and production yield and quality. However, the long-term use of these additives, including 3-NOP, raises concerns due to the limited evidence available regarding their safety and efficacy over extended periods. Comprehensive studies are essential to assess the impact of these additives on livestock health and their environmental effects. In addition, there should be a greater focus on precision livestock farming. This approach can utilize phenotypic data to enhance EME estimation and improve the quality of diets through precise analysis. By leveraging precision systems, researchers can implement modifications that optimize animal nutrition, ultimately leading to more effective methane reduction strategies. Moreover, implementing more manageable and precise methane measurement systems will allow for a more accurate selection of individuals that emit less methane, enhancing breeding programs focused on low-emission livestock. Integrating these areas of research will be crucial for advancing our understanding of EME and developing sustainable solutions in the livestock sector. Collaboration among scientists, policymakers, and industry stakeholders will further enhance the effectiveness of these efforts.

## Figures and Tables

**Table 1:** Bibliographic search strings for the text mining analysis on enteric methane emission in the livestock sector carried out on titles, abstracts, and keywords of peer-reviewed literature in English published between 1986 and May 2024.

Search Words	Original No. of Records
Enteric Methane AND Emission	1711
Enteric Methane AND Ruminants	742
Enteric Methane AND Cow	612
Enteric Methane AND Sheep OR Goat	242
Enteric Methane AND Additive	221
Enteric Methane AND Buffalo	68
Enteric Methane AND Microbiome OR Microbiota	28
Total	3624

**Table 2:** Relationships between the most pertinent terms (TF-IDF ≥ 9.5) and the other terms in the corpus of 1294 records.

Words (TF-IDF ≥ 9.5)	Associated Words (Grade of Correlation ≥ 0.4)
Cow	Lactat (0.47)
Model	Error (0.46)
Digest	Nutrient (0.42)
Energi	Gross (0.46); Metaboliz (0.41)
Graze	Pastur (0.43)
Increas	Linear (0.45)
Measur	Chamber (0.41)
Predict	Error (0.50); Equat (0.47); Extant (0.42)

The correlation grade is expressed in parentheses. The minimum grade of correlation was set as ≥0.4. Because the text-stemming method reduced the words to their origins, words could be partially cut.

**Table 3:** Topics obtained from latent Dirichlet allocation (LDA) analysis.

Topic	Label of Topic	Acronyms	No. of Records per Topic (%)	Year of First Publication
1	Methane emission-animal	ME-A	100 (7.73%)	1998
2	Extensive farming system	EFS	120 (9.27%)	2005
3	In vivo measurement system	MS	127 (9.81%)	2001
4	Supplement and additive	SA	142 (10.97%)	2006
5	Ruminal fermentation	RF	132 (10.20%)	2006
6	Diet composition	DC	174 (13.45%)	2005
7	Dairy production	DP	119 (9.20%)	2005
8	Prediction model	PM	146 (11.28%)	2005
9	Greenhouse gas emission from livestock	GHGL	234 (18.08%)	1986

**Table 4:** Continental animal CH_4_ measurement studies published from 1994 to 2018, adapted by [32].

Continent	Studies (%)	Species ^1^	EME Technique ^2^
Europe	32	Cattle, goat, sheep, and other	RC, SF_6_, GF, and other
Oceania	23	Cattle, sheep, and other	RC, SF_6_, GF, and other
North America	20	Cattle, goat, sheep, and other	RC, SF_6_, and GF
Asia	15	Buffalo, cattle, goat, sheep, and other	RC, SF_6_, GF, and other
South America	9	Cattle, goat, and sheep	RC, SF_6_, and other
Africa	1	Cattle and goat	SF_6_ and other

^1^ Other includes other species: alpaca, llama, deer, and bison; RC: respiration chamber; SF_6_: sulfur hexafluoride; GF: GreenFeed; ^2^ other includes other techniques: face mask, sniffer, laser methane detector, and portable accumulation chamber.

## Data Availability

Data sharing not applicable.

## Supplementary Materials

The following supporting information can be downloaded at: https://www.mdpi.com/article/10.3390/ani14213158/s1.
