# Supplementary material for: Enteric Methane Emission in Livestock Sector: Bibliometric Research from 1986 to 2024 with Text Mining and Topic Analysis Approach by Machine Learning Algorithms
Source: Animals (Basel). 2024 Nov 4;14(21):3158. doi: 10.3390/ani14213158 (PMC11545165; doi:10.3390/ani14213158)
Supplement: Supplementary file 1 [file animals-14-03158-s001.zip › animals-3244258-supplementary.pdf]

Below is a more detailed description of these packages from a practical point of view:

tm

The tm package is foundational for text mining applications in R. It provides a comprehensive framework for handling and processing text data.

- **Practical Use**:

    Corpus Creation: Using VCorpus(), we can create a volatile corpus that holds text data in a structured format, allowing for efficient manipulation.

    Data Transformation: Functions like tm_map() enable us to apply various transformations, such as cleaning text, removing stopwords, and converting text to lowercase. This preprocessing is essential to ensure that the text data is in the right format for analysis.

Snowball

This package is specifically designed for stemming, which is the process of reducing words to their root forms.

- **Practical Use**:

    The function stemDocument() is utilized to stem words within the corpus. This step is crucial in consolidating different word forms (e.g., "emission," "emissions," "emit") into their base form ("emiss"), which simplifies analysis and enhances the accuracy of results.

ggplot2

ggplot2 is a powerful visualization package based on the Grammar of Graphics, allowing users to create complex and aesthetically pleasing graphics.

- **Practical Use**:

    Through functions like geom_bar() for bar plots and scale_fill_gradient() for color gradients, we visualize word frequency data. This aids in interpreting results, making it easier to identify trends and patterns in the text data.

dplyr

This package is focused on data manipulation and provides a set of functions that simplify data frame operations.

- **Practical Use**:

    The filter() function allows for sub setting data based on specific criteria, which we used to isolate significant words with weights exceeding a certain threshold. This targeted approach helps in focusing on the most relevant terms during analysis.

tidyverse

The tidyverse is a collection of R packages designed for data science, offering tools for data manipulation and visualization.

- **Practical Use**:
    - Within the tidyverse, we leveraged filter() and ggplot() for data management and visualization. This integration enhances the workflow by providing consistent syntax and a cohesive approach to handling and visualizing data.

topicmodels

This package provides the functionality needed to fit topic models to text data, including Latent Dirichlet Allocation (LDA).

- **Practical Use**:

    The LDA() function is critical for performing topic analysis. We defined the number of topics to be extracted and utilized Gibbs sampling to iteratively estimate topic distributions. This approach enabled us to uncover underlying themes in the corpus effectively. The topicmodels package was employed to perform topic analysis through Latent Dirichlet Allocation (LDA). We used this package to identify and classify themes present in the corpus, setting specific parameters for Gibbs sampling and determining the number of topics to explore

Practical Description of Dirichlet Allocation and Bayesian Probabilistic Techniques

Latent Dirichlet Allocation (LDA) is a generative model used for topic modelling, identifying hidden topics in a document set under the assumption that each document is a mixture of topics, each characterized by a word distribution.

Practical Implementation Steps:

Data Preparation: Created a Document-Term Matrix (DTM) using the tm package to represent word frequencies.

Model Specification: Specified the number of topics (k = 9) using the topicmodels package.

Gibbs Sampling: Applied Gibbs sampling to iteratively estimate topic distributions for each document.

Output Analysis: Analysed generated topic-word distributions to identify main themes.

Visualization: Visualized results using ggplot2 for easier interpretation.

Bayesian Probabilistic Techniques

Bayesian methods employ Bayes' theorem to update hypothesis probabilities based on new evidence. In the context of LDA, these techniques help infer topic and word distributions.

Practical Application:

Dirichlet Priors: Set initial beliefs for topic and word distributions, adjusting these beliefs after running LDA based on observed data.

Inference: Used Gibbs sampling to draw samples from posterior distributions, refining topic assignments.

Interpretation: This Bayesian approach provided insights into the likelihood of topics and their associated words, enhancing our understanding of the corpus.
